# A randomised controlled trial of energetic activity for depression in young people (READY): a multi-site feasibility trial protocol

**DOI:** 10.1186/s40814-020-00734-7

**Published:** 2021-01-04

**Authors:** N. Howlett, L. Bottoms, A. Chater, A. B. Clark, T. Clarke, L. David, K. Irvine, A. Jones, J. Jones, S. E. Mengoni, J. Murdoch, M. Pond, S. Sharma, E. J. Sims, D. A. Turner, D. Wellsted, J. Wilson, S. Wyatt, D. Trivedi

**Affiliations:** 1 Department of Psychology, Sport, and Geography, University of Hertfordshire, College Lane, Hatfield, Herts AL10 9AB UK; 2 Institute for Sport and Physical Activity Research (ISPAR), School of Sport Science and Physical Activity, Faculty of Health, Education, Sport and Social Science, University of Bedfordshire, Polhill Avenue, Bedford, MK41 9EA UK; 3 Norwich Clinical Trials Unit, University of East Anglia, Norwich, Norfolk NR4 7TR UK; 4 Norfolk and Suffolk NHS Foundation Trust, Hellesdon Hospital, Drayton High Road, Norwich, NR6 5BE UK; 5 Centre for Health Services and Clinical Research, University of Hertfordshire, College Lane, Hatfield, Herts AL10 9AB UK; 6 Norwich Medical School, University of East Anglia, Norwich, Norfolk NR4 7TJ UK; 7 Centre for Research in Public Health and Community Care, University of Hertfordshire, College Lane, Hatfield, Herts AL10 9AB UK

**Keywords:** Depression, Low mood, Young people, Exercise, Physical activity, Behaviour change, Feasibility trial

## Abstract

**Background:**

Prevalence of depression is increasing in young people, and there is a need to develop and evaluate behavioural interventions which may provide benefits equal to or greater than talking therapies or pharmacological alternatives. Exercise could be beneficial for young people living with depression, but robust, large-scale trials of effectiveness and the impact of exercise intensity are lacking. This study aims to test whether a randomised controlled trial (RCT) of an intervention targeting young people living with depression is feasible by determining whether it is possible to recruit and retain young people, develop and deliver the intervention as planned, and evaluate training and delivery.

**Methods:**

The design is a three-arm cluster randomised controlled feasibility trial with embedded process evaluation. Participants will be help-seeking young people, aged 13–17 years experiencing mild to moderate low mood or depression, referred from three counties in England. The intervention will be delivered by registered exercise professionals, supported by mental health support workers, twice a week for 12 weeks. The three arms will be high-intensity exercise, low-intensity exercise, and a social activity control. All arms will receive a ‘healthy living’ behaviour change session prior to each exercise session and the two exercise groups are energy matched. The outcomes are referral, recruitment, and retention rates; attendance at exercise sessions; adherence to and ability to reach intensity during exercise sessions; proportions of missing data; adverse events, all measured at baseline, 3, and 6 months; resource use; and reach and representativeness.

**Discussion:**

UK National Health Service (NHS) policy is to provide young people with advice about using exercise to help depression but there is no evidence-based exercise intervention to either complement or as an alternative to medication or talking therapies. UK National Institute for Health and Care Excellence (NICE) guidelines suggest that exercise can be an effective treatment, but the evidence base is relatively weak. This feasibility trial will provide evidence about whether it is feasible to recruit and retain young people to a full RCT to assess the effectiveness and cost-effectiveness of an exercise intervention for depression.

**Trial registration:**

ISRCTN, ISRCTN66452702. Registered 9 April 2020.

## Introduction

### Background and rationale

The prevalence of depression amongst young people is high worldwide and recorded rates have increased significantly since the 1980s [1–3]. Depression levels rise sharply as children transition into adolescence, with estimates of depression reported to be between 4 and 11% in mid-to-late adolescence (15–16 years old) and up to 20% by late adolescence (up to 18 years old) [3–5], although prevalence estimates vary widely across studies and countries [4]. A noted trend is the rise in prevalence of depression amongst adolescent females compared to males, estimated at 2:1 [1–4, 6–10]. By 13–15 years, more girls are diagnosed with depression compared to boys [11]. The reasons for this trend are not fully understood but may be related to hormonal changes during puberty, or the tendency for greater internalisation of emotion in girls [4]. These findings are supported by a recent large-scale survey in England (aged 11–15) which found poorer emotional health and wellbeing amongst adolescent girls compared to boys [12, 13]. Evidence also suggests that there are significant disparities in mental health experiences amongst specific groups of young people. For example, those identifying as lesbian, gay, bisexual, and transgender (LGBT) report experiencing depression and anxiety, suicidality, and self-harm at considerably higher rates than heterosexual young people of a similar age, influenced by factors such as bullying and perceived stigma [14, 15]. Available research also points to higher rates of symptoms amongst youth from Black and Asian minority ethnic (BAME) backgrounds [16, 17]. This reflects a complex interplay of the clustering of multiple disadvantages, and the concept of double minorities highlights how factors such as race/ ethnicity and gender identity can further compound mental well-being [18].

Recent research suggests that young people who seek help benefit from contact with mental health services. The UK-based ROOTS longitudinal cohort study found that contact with mental health services by 14-year-olds with depression reduced the likelihood of depression by age 17 [7]. This is particularly important as it is known that many young people with depression do not access mental health services, estimated at 34–56% internationally [7], or delay seeking help, increasing the duration or risk of recurrent episodes [7]. There are, however, concerns regarding the use of antidepressant drugs for those younger than 18 years. Recent meta-analyses suggest that antidepressants for children and adolescents do not generally perform better than placebos [19] and pose an increased risk for suicidal thoughts and aggressive behaviour [20]. For this reason, it is important to identify a range of effective non-pharmacological alternatives to increase the available options for treatment.

One such alternative is psychological approaches that include cognitive behavioural therapy (CBT) and interpersonal psychotherapy. CBT has been shown to be effective in lowering risk of depression in children and adolescents living with subclinical depression [21]. Interpersonal psychotherapy is also beneficial for adolescents living with depression, but few trials compare efficacy with alternative treatments [22]. When treatment outcomes were compared across different therapy types, there was no evidence that one was superior and only 8–27% of the 11–17-year-old participants completed the recommended number of sessions [23]. These findings are echoed by the latest national data suggesting that only 36% of people complete the full Improving Access to Psychological Therapies treatment [24]. Given the cost of delivering individual face-to-face psychological therapies and the low completion rates, there is a need for alternative approaches which promote changes in health behaviours such as exercise. Promoting aerobic exercise can also provide additional improvements in cholesterol and blood lipids, blood pressure, metabolic syndrome, overweight and obesity, and bone mineral density [25], and health-related quality of life [26].

There is clear evidence for the effectiveness of exercise to support adults living with depression, with a recent meta-analysis of exercise finding large significant effects on depression [27]. However, for young people living with depression, the evidence base is scarce and evidence quality is poor. A Cochrane review in 2006 [28] and subsequent systematic reviews in 2013, 2016, and 2018 [29–31] examined the effects of exercise interventions in reducing depression and anxiety in children and adolescents. Larun et.al [28]. and Brown et.al [29]. found a small effect in favour of exercise interventions compared to control groups. Carter and colleagues [30] found a moderate effect on depressive symptoms in clinical samples. Bailey et.al [31]. also reported that exercise was an acceptable and feasible intervention for this target group, with low dropout. However, the low quality of the studies, the small number of studies, small sample sizes, and diversity of participants, interventions, and methods of measurement limit the ability to draw firm conclusions. There is a need for well-powered and robust trials with help-seeking young people in real-world treatment settings that explore effectiveness alongside the mechanisms by which exercise help and what impact intensity of exercise has on depression.

There are several mechanisms that have been proposed to explain the many ways by which exercise may be beneficial in the management of depression [32]. Some of these might be via social mechanisms; exercise participation can provide a diversion from depressive thoughts, opportunities to learn new skills, and increased socialisation [33]. In addition, there may be physiological mechanisms; exercise is associated with promoting the release of endorphins and serotonin which can improve mood [34]. Further, inflammation has been identified as a potential contributor to the development of depression [35], suggesting that anti-inflammatory strategies, such as regular exercise [36], may be effective at preventing and managing depressive symptoms.

The optimal intensity of exercise required has not been established, and this information is critical when determining exercise on prescription for depression. Moderate levels of exercise have been shown to increase myokines (proteins released by muscle cells) which could have a positive impact on inflammation [37] and hence depression. In recent years, it has come to light that performing very short bursts of high-intensity exercise (30 s) followed by 30 s of rest repeated for 4 min has produced increased fat oxidation and increased maximal oxygen uptake [38]. Furthermore, research has suggested that high-intensity interval training (HIIT) can promote anti-inflammatory effects during recovery [39] and therefore could be beneficial for diseases with an inflammatory response [40, 41]. There remains considerable uncertainty about the extent to which exercise intensity is related to benefit for depression.

Overall, young people are underserved by child and adolescent mental health services and at high risk of continued mental health problems into adulthood. There is an urgent need to offer feasible, acceptable, and cost-effective treatment options for this group, given the limitations and potential risks of pharmacotherapy treatment and long waiting times for psychological support. NICE clinical guidance [42] recommends that adolescents with depression should be offered advice on the benefits of regular exercise and encouraged to follow a structured and supervised exercise programme of up to three sessions a week, 45–60 min, for 10–12 weeks. Consequently, current NHS policy and practice regarding exercise is to provide guidance rather than a structured and supervised exercise intervention. However, the NICE guidance is based on weak evidence (level IV) due to a lack of high-quality studies in this area. The current study will ascertain the feasibility of conducting a large, adequately powered, high-quality RCT with help-seeking young people with depression. Additionally, given pressure on NHS budgets and the many competing uses of NHS resources, it is not sufficient to show that an intervention is effective, we must also show it represents good value for money compared to other potential uses of these resources, i.e., that it is cost-effective. Our research will also add important knowledge regarding the feasibility of partnership working across the NHS and local community organisations, such as Active Partnerships, in areas with diverse populations and deprivation, to improve mental health support for young people [9, 43].

In summary, the existing evidence suggests that exercise may be a promising and acceptable intervention for young people living with depression. There is clear need to test the feasibility of running a high-quality trial in this area, in terms of recruitment and retention of young people, development, training, and delivery of the intervention as planned, and provide data to inform a full trial.

### Objectives

The objective is to establish the feasibility of conducting a full RCT with embedded process evaluation and parallel cost-effectiveness, with user and stakeholder input in multiple English counties (Hertfordshire, Bedfordshire, Norfolk). It will determine whether it is possible to recruit and retain young people to the trial, develop and deliver the intervention as planned, evaluate the training and delivery of the intervention, and will provide data to inform the design of the main trial (e.g. required sites and sample size).

### Trial design

A three-arm, multi-site, 12-week cluster randomised controlled feasibility trial with 6-month follow-up.

## Methods

### Participants, interventions, and outcomes

#### Study setting

The READY feasibility trial is taking place in Hertfordshire, Bedfordshire, and Norfolk with GP involvement. The intervention will be delivered by registered exercise professionals (REP), employed by local physical activity providers (e.g. Watford FC Community and Sports Trust, Active Luton, Norwich City FC Community Sports Foundation), in local community venues (e.g. sports facilities and community halls).

#### Eligibility criteria

The inclusion criteria includes help-seeking young people aged 13–17 years with a Children’s Depression Inventory (CDI 2[44]) score between 17 and 36 (mild to moderate symptoms), who understand their role in the trial and are able to complete trial activities, who can get to the intervention venue, consent to participate, with the consent form being completed for parent/carer for under 16 s and consent of parent/carer to provide data and take part in the study, and both young person and parent/carer able to complete the questionnaires in English. Current treatment with antidepressants or other drug(s) or psychological therapy is allowed.

The exclusion criteria includes being considered unsuitable by the clinician screening for eligibility, their current treatment or co-morbid conditions presenting contraindications to engaging in the RCT or exercise, active psychosis, significant substance abuse, self-harm, or suicidal ideation presenting significant risk (assessed as part of the Development and Wellbeing Assessment (DAWBA)).

#### Interventions

The intervention sessions will run twice a week for 12 weeks, with a 15–20 min ‘healthy living’ behaviour change portion of the session (in all arms), followed by the exercise or social portion of the sessions. The proposed exercises are based on research by Weston et al. [45] who developed an exercise protocol from qualitative data collected at focus groups from adolescent school children. It incorporates activities which will appeal to both males and females and has been designed with the consultation of a range of young people.

##### High-intensity exercise arm

This is composed of high-intensity exercises of alternating training sessions [45, 46] (e.g. basketball, football, boxing drills—see Table 1 for full breakdown) beginning with a 10-min warm up, culminating with a 5-min whole body cool down. Young people will perform four repetitions of 45 s of maximal effort exercise (> 90% predicted maximal heart rate) with 90 s rest in between each repetition (approximately 9 min). This will increase by 2 min and 15 s every 2 weeks (e.g. one repetition of 45 s of exercise and 90 s rest) for the first 6 weeks and 4 min 30 s (e.g. two repetitions of 45 s of exercise and 90 s rest) in the last 6 weeks. The final 2 weeks will be 12 repetitions (27 min of exercise). Heart rate monitors will be used to tailor each person’s maximum intensity and measure exertion in each session [47].

**Table 1:** Exercise session outline

Week	Session number	High-intensity exercise component	Low-intensity exercise component
1	1	10 min warm up Boxing (4 reps of 45 s and 90 s rest) 5 min cool down **Total time: 24 min**	10 min warm up Walking football (7.5 min halves) *2 min rest at half time* 5 min cool down **Total time: 32 min**
	2	10 min warm up Circuit training to music (4 reps of 45 s and 90 s rest) 5 min cool down **Total time: 24 min**	10 min warm up Walking netball (7.5 min halves) *2 min rest at half time* 5 min cool down **Total time: 32 min**
2	3	10 min warm up Football (4 reps of 45 s and 90 s rest) 5 min cool down **Total time: 24 min**	10 min warm up Walking basketball (7.5 min halves) *2 min rest at half time* 5 min cool down **Total time: 32 min**
	4	10 min warm up Basketball (4 reps of 45 s and 90 s rest) 5 min cool down **Total time: 24 min**	10 min warm up Walking dodgeball (7.5 min halves) *2 min rest at half time* 5 min cool down **Total time: 32 min**
3	5	10 min warm up Choice/combination (5 reps of 45 s and 90 s rest) 5 min cool down **Total time: 26 min 15 s**	10 min warm up Walking choice (9 min halves) *2 min rest at half time* 5 min cool down **Total time: 35 min**
	6	10 min warm up Boxing (5 reps of 45 s and 90 s rest) 5 min cool down **Total time: 26 min 15 s**	10 min warm up Walking choice (9 min halves) *2 min rest at half time* 5 min cool down **Total time: 35 min**
4	7	10 min warm up Circuit training to music (5 reps of 45 s and 90 s rest) 5 min cool down **Total time: 26 min 15 s**	10 min warm up Walking choice (9 min halves) *2 min rest at half time* 5 min cool down **Total time: 35 min**
	8	10 min warm up Football (5 reps of 45 s and 90 s rest) 5 min cool down **Total time: 26 min 15 s**	10 min warm up Walking choice (9 min halves) *2 min rest at half time* 5 min cool down **Total time: 35 min**
5	9	10 min warm up Basketball (6 reps of 45 s and 90 s rest) 5 min cool down **Total time: 28 min 30 s**	10 min warm up Walking choice (10.5 min halves) *2 min rest at half time* 5 min cool down **Total time: 38 min**
	10	10 min warm up Choice/combination (6 reps of 45 s and 90 s rest) 5 min cool down **Total time: 28 min 30 s**	10 min warm up Walking choice (10.5 min halves) *2 min rest at half time* 5 min cool down **Total time: 38 min**
6	11	10 min warm up Boxing (6 reps of 45 s and 90 s rest) 5 min cool down **Total time: 28 min 30 s**	10 min warm up Walking choice (10.5 min halves) *2 min rest at half time* 5 min cool down **Total time: 38 min**
	12	10 min warm up Circuit training to music (6 reps of 45 s and 90 s rest) 5 min cool down **Total time: 28 min 30 s**	10 min warm up Walking choice (10.5 min halves) *2 min rest at half time* 5 min cool down **Total time: 38 min**
7	13	10 min warm up Football (8 reps of 45 s and 90 s rest) 5 min cool down **Total time: 33 min**	10 min warm up Walking choice (13.5 min halves) *2 min rest at half time* 5 min cool down **Total time: 44 min**
	14	10 min warm up Basketball (8 reps of 45 s and 90 s rest) 5 min cool down **Total time: 33 min**	10 min warm up Walking choice (13.5 min halves) *2 min rest at half time* 5 min cool down **Total time: 44 min**
8	15	10 min warm up Choice/combination (8 reps of 45 s and 90 s rest) 5 min cool down **Total time: 33 min**	10 min warm up Walking choice (13.5 min halves) *2 min rest at half time* 5 min cool down **Total time: 44 min**
	16	10 min warm up Boxing (8 reps of 45 s and 90 s rest) 5 min cool down **Total time: 33 min**	10 min warm up Walking choice (13.5 min halves) *2 min rest at half time* 5 min cool down **Total time: 44 min**
9	17	10 min warm up Circuit training to music (10 reps of 45 s and 90 s rest) 5 min cool down **Total time: 37 min 30 s**	10 min warm up Walking choice (16.5 min halves) *2 min rest at half time* 5 min cool down **Total time: 50 min**
	18	10 min warm up Football (10 reps of 45 s and 90 s rest) 5 min cool down **Total time: 37 min 30 s**	10 min warm up Walking choice (16.5 min halves) *2 min rest at half time* 5 min cool down **Total time: 50 min**
10	19	10 min warm up Basketball (10 reps of 45 s and 90 s rest) 5 min cool down **Total time: 37 min 30 s**	10 min warm up Walking choice (16.5 min halves) *2 min rest at half time* 5 min cool down **Total time: 50 min**
	20	10 min warm up Choice/combination (10 reps of 45 s and 90 s rest) 5 min cool down **Total time: 37 min 30 s**	10 min warm up Walking choice (16.5 min halves) *2 min rest at half time* 5 min cool down **Total time: 50 min**
11	21	10 min warm up Boxing (12 reps of 45 s and 90 s rest) 5 min cool down **Total time: 42 min**	10 min warm up Walking choice (2 × 10 min and 2 × 9 min quarters) *1 min rest between* 5 min cool down **Total time: 57 min**
	22	10 min warm up Circuit training to music (12 reps of 45 s and 90 s rest) 5 min cool down **Total time: 42 min**	10 min warm up Walking choice (2 × 10 min and 2 × 9 min quarters) *1 min rest between* 5 min cool down **Total time: 57 min**
12	23	10 min warm up Football (12 reps of 45 s and 90 s rest) 5 min cool down **Total time: 42 min**	10 min warm up Walking choice (2 × 10 min and 2 × 9 min quarters) *1 min rest between* 5 min cool down **Total time: 57 min**
	24	10 min warm up Basketball (12 reps of 45 s and 90 s rest) 5 min cool down **Total time: 42 min**	10 min warm up Walking choice (2 × 10 min and 2 × 9 min quarters) *1 min rest between* 5 min cool down **Total time: 57 min**

On arrival, participants get changed. They will also put the heart rate monitor on, and this will be demonstrated in an earlier session

*High-intensity interval training exercises*

Boxing

Reps can include any of the following:

• Ten jabs, followed by running to the end of the sports hall and back

• Ten hooks, followed by five squat thrusts

• Fast upper cuts

• Ten jabs, followed by five-star jumps

• Fast jabs

• Ten side steps, followed by running to the end of the sports hall and back

• Five combination punches (hook and jab), followed by running to the end of the sport hall and back

• Ten of favourite punch action, followed by ten tuck jumps

Circuit training to music

• Full star jumps

• Tuck jumps

• Stationary high knees runs

• Jumping with one hand in the air

• Hop on one leg

• Push ups

• Sit ups

• High leg kicks

• Fast side kicks

• Fast side to side twists

Football

• Ten toe touches, followed by running to a cone and back

• Running up to kick the ball ten times, followed by five burpees

• Sprinting around cones in the sports hall

• Kicking a football into a goal then running to end of the sports hall and back. Performing fast feet movements through cones then running to end of the sport hall and back

• Jumping up to head a football five times then running to the end of the sports hall and back

• Running around the sports hall following a square or diagonal course

Basketball

• Receiving and returning a chest pass, followed by running to a cone and back

• Running around in a square and receiving and returning a bounce pass on one corner of the square

• Bouncing a ball five times then running to the end of the hall and back

• Receiving a shoulder pass, followed by running to a cone and back

##### Low-intensity exercise arm

This is composed of low-intensity exercise of alternating training sessions [48] (e.g. walking football, walking netball). These activities elicit a heart rate between 40 and 50% maximal effort based on the activity compendium [49]. The sessions will follow the same warm up and cool down as the high intensity, but the overall exercise session will be longer (to energy match to high intensity). The first 2 weeks will start at 15 min of exercise with a 2-min break in the middle. This will increase by 3 min every 2 weeks for the first 6 weeks, and 6 min every 2 weeks for the second 6 weeks. The last 2 weeks will therefore consist of 38 min of exercise (including the break).

##### Energy matching

A pilot study was conducted to ensure that the low- and high-intensity arms will exert an equivalent amount of energy, using a standardised protocol [Bottoms, Howlett, Chater, Jones, Jones, Wyatt, et al. Energy matching of a high intensity exercise protocol with a low intensity exercise protocol in adolescents. Under Review.]. This will ensure that any differences between groups will be down to intensity of exercise rather than the amount of energy expended. Following walking on a treadmill at a comfortable speed for 5 min, 24 participants (15 boys and 9 girls) completed the low-intensity exercise protocol for 10 min (walking football), rested until their heart rate had returned to baseline, and then completed the high-intensity interval exercise protocol (boxing), for 9 min. Nine minutes of the high-intensity exercise (e.g. four repetitions of 45 s of exercise and 90 s rest) was the equivalent of approximately 12 min of continuous low-intensity exercise.

##### Social control arm

Social activities will include board and team games (e.g. giant Jenga), and group discussions, with the exact activities agreed upon by the group. The purpose of these control activities is to provide a comparative length of time (to the exercise groups) and social context, which does not involve exercise, to estimate any potential social benefits for depression of the two exercise conditions. The Healthy Living session will be the same as other trial arms to avoid introducing variables other than supervised exercise sessions into the study design. Young people in all three arms will be encouraged to engage in physical activity after the intervention ends to maintain their exercise levels.

##### Behaviour change and maintaining engagement

This model of delivery is based on feedback from young people, the team’s experience, and the importance of adherence [50].

Behaviour change techniques (BCT [51];) have been derived using the Behaviour Change Wheel approach, incorporating the COM-B system [52] and the Theoretical Domains Framework (TDF [53]) used as a theoretical base for the ‘Healthy Living’ component of the intervention, e.g. [54]. There are two main objectives of the 15–20 min Healthy Living sessions: (1) to help ensure the young people attend the sessions and engage with the intervention; and (2) to encourage the sustainability of physical activity engagement after the intervention has ended.

These sessions will address key BCTs, delivered through the REPs using Motivational Interviewing and Health Coaching, to promote engagement and enable young people to drive their own goals, learning, and behaviour. The BCTs will use theoretical drivers from the COM-B and TDF to target barriers and facilitators to Capability, Opportunity, and Motivation (see Table 2 for full mapping of theoretical content). Barriers, highlighted by our consultations with young people, include a lack of ‘head space’ or stamina for exercise (Psychological and Physical Capability), lack of social support (Social Opportunity), negative beliefs about exercise (Reflective Motivation), and emotions that lead to avoidance (Automatic Motivation). Potential facilitators were suitable environments (Physical Opportunity) to increase access, manageable sized groups (6–10 young people) and peer support (Social Opportunity), setting goals and increasing positive expectations about exercise (Reflective Motivation). Motivational interviewing (MI) and health coaching will be used to deliver BCTs that will target these barriers and facilitators to enhance intrinsic motivation [57, 58], and address potential depression-related barriers using aspects of behavioural activation, such as activity scheduling and reducing avoidance.

**Table 2:** Intervention content mapped from the COM-B to the TDF, intervention functions, policy categories, and behaviour change techniques (BCTs) (based on [51, 53, 55, 56]).

COM-B	Theoretical domain	Intervention function	Policy category	BCTs	Intervention content	Mode of delivery
**CAPABILITY** (Psychological)	Knowledge	Education	Service provision	4.1 Instruction on how to perform the behaviour	The REP will provide instruction on how to perform the activities involved in each session prior to exercising.	Face-to-face group exercise session
				9.1 Credible Source	The REP will be well qualified to deliver exercise sessions, with strong experience and expertise, and will advocate performing exercise during the structured sessions and then promote sustainable physical activity habits after the intervention	Face-to-face group exercise and behaviour change sessions
				2.6 Biofeedback	Participants will be using heart rate monitors to help guide physical effort during the exercise sessions	Face-to-face group exercise session
				5.1 Information about health consequences	Group discussions about health consequences of physical activity	Face-to-face group and behaviour change sessions
	Memory, attention and decision processes	Education	Service provision	1.2 Problem Solving (including relapse prevention)	Group discussions of barriers and solutions to challenges in attending and engaging with the exercise sessions. Towards the end of the intervention, group discussions will prospectively explore potential barriers and solutions to participating in more physical activity after the intervention is finished.	Face-to-face group and behaviour change sessions
	Behavioural regulation	Education	Service provision	2.3 Self-monitoring of behaviour	Encouragement to self-monitor their behaviour with a physical activity diary or a step counter on their phone after the intervention is finished.	Face-to-face group and behaviour change sessions
**CAPABILITY** (Physical)	Skills	Training	Service provision	6.1 Demonstration of the behaviour	The REP will demonstrate the activities involved in each session prior to exercising	Face-to-face group exercise session
				8.7 Graded tasks	The sessions will build in duration (and intensity depending on heart rate feedback), fortnightly, over the 12 week intervention	Face-to-face group exercise session
				8.1 Behavioural practice/rehearsal	The young people will be practising activities at high or low intensity in every session that they attend.	Face-to-face group exercise session
				8.3 Habit formation	Encourage making physical activity habits long term (after the intervention) by trying to perform it in the same context (this could be time/day, activity, or environment)	Face-to-face group and behaviour change sessions
				8.6 Generalisation of the target behaviour	Group discussions on the transition from the exercise classes to wider physical activity after the intervention is finished	Face-to-face group and behaviour change sessions
**MOTIVATION** (Reflective)	Beliefs about capabilities	Persuasion	Service provision	15.3 Focus on past success	Group discussion of examples of past success in terms of previous attendance and engagement with exercise (e.g. school, club, hobbies). Towards the end of the intervention, group discussion will explore of examples of past success in terms of being active in their own time (e.g. hobbies, walking, cycling) to encourage sustainable physical activity habits	Face-to-face group and behaviour change sessions
				15.1 Verbal persuasion about capability	Provide positive talk about the young peoples’ ability to attend and participate in the exercise sessions regularly. Towards the end of the intervention, positive talk will focus on the young peoples’ ability to engage in regular physical activity after the intervention is finished	Face-to-face group and behaviour change sessions
	Beliefs about consequences	Education	Service provision	9.2 Pros and Cons	Group discussions regarding the pros and cons of attending and participating in the exercise sessions. Towards the end of the intervention, group discussions will explore the pros and cons of physical activity after the intervention is finished	Face-to-face group and behaviour change sessions
				5.3 Information about social and environmental consequences	Towards the end of the intervention, group discussions will explore the social consequences of physical activity and the environmental consequences of active travel (e.g. cycling vs car use)	Face-to-face group and behaviour change sessions
				5.6 Information about emotional consequences	Group discussions about the emotional consequences of the structured exercise during the sessions. Towards the end of the intervention, group discussions will explore the emotional consequences of participating in physical activity after the intervention is finished.	Face-to-face group and behaviour change sessions
	Goals	Education	Service provision	1.1 Goal setting (behaviour) 1.4 Action planning	The group will discuss setting detailed plans about incorporating attending the sessions into their weekly routines. Towards the end of the intervention, group discussions will explore setting detailed plans about incorporating physical activity into their weekly routines after the intervention (including signposting local opportunities)	Face-to-face group and behaviour change sessions
				1.5 Review behaviour goal	Encourage reviewing their goals related to the exercise sessions. Towards the end of the intervention, group discussions will encourage reviewing their goals related to physical activity after the intervention is finished	Face-to-face group and behaviour change sessions
**MOTIVATION** (Automatic)	Reinforcement	Incentivisation	Service provision	10.4 Social reward	Young people will be rewarded verbally, providing positive reinforcement, for turning up and participating in the sessions.	Face-to-face group exercise session
				10.9 Self-reward	Encourage rewarding themselves for making progress and/or meeting their goals of engagement with the exercise sessions.	Face-to-face group and behaviour change sessions
				10.7 Self-incentive	Towards the end of the intervention, encourage rewarding themselves for making progress and/or meeting their goals for physical activity after the intervention sessions in the future	Face-to-face group and behaviour change sessions
		Education		5.4 Monitoring of emotional consequences	Encouragement to make mental or physical notes about how they feel during or after the exercise sessions	Face-to-face group and behaviour change sessions
**OPPORTUNITY** (Physical)	Environmental context and resources	Environmental restructuring	Environmental/social planning	12.1 Restructuring the physical environment	The sessions are being added to the young peoples’ environment, for free	Face-to-face group exercise session
				7.1 Prompts/cues	Encouraging young people to leave reminders to prepare for and attend the exercise sessions	Face-to-face group and behaviour change sessions
**OPPORTUNITY** (Social)	Social influences	Environmental restructuring	Environmental/social planning	12.2 Restructuring the social environment	The sessions are being added to the young peoples’ environment, with peers of similar ages, living with depression to provide social support	Face-to-face group exercise session
		Enablement	Service provision	3.1 Social support (unspecified) 3.2 Social support (practical) 3.3 Social support (emotional)	Group discussions of how to ask for and use social support from friends/family/guardians to attend and engage in the exercise sessions if needed. Towards the end of the intervention, group discussions will explore how to build social support for physical activity after the intervention is finished	Face-to-face group and behaviour change sessions
				13.1 Identification of self as role model	Towards the end of the intervention, encourage discussions of how they should think of themselves as role models for exercise for others (friends, family)	Face-to-face group and behaviour change sessions

##### Deliverers and training

The intervention sessions will be delivered primarily by a REP, with a minimum of level 3 qualifications or equivalent, contracted to local community sport and physical activity organisations. A mental health support worker (MHSW) (e.g. an assistant psychologist) will assist the REP in delivering the sessions and will be employed by the local NHS trust. Prior to attending training deliverers will complete the online Good Clinical Practice training from the NIHR [59]. The REPs and MHSWs will then attend 3 days of training (spread over approximately 4 weeks): day 1 will focus on good clinical practice, running the exercise sessions, heart rate monitoring, and research skills related to outcome assessment and data management; the second day will focus on encouraging attendance, adherence, and engagement with the sessions, and longer-term behaviour change; the third day will focus on consolidating the exercise, delivery, behaviour change, and research skill, and mental health training for physical activity promotion.

The training day on behaviour change will cover the ‘healthy living’ behaviour change session content and communication skills using motivational interviewing [60], and how to deliver the BCTs with an emphasis on expressing empathy and being client-focused [61]. This training will highlight the need to engage the young people in the discussions, resist telling them what to do (the righting reflex), allowing focus on what is desired and achievable, to understand their perspective, evoke a sense of empowerment, ensure they feel supported, and have a plan going forward [62]. Core communication skills to support effective group discussions [57, 63] such as RULE (Resist the righting reflex; Understand your client’s motivation; Listen to your client; Empower your client) and OARS (Open-ended questions, Affirmations, Reflective listening, Summaries) will be covered and linked to the delivery of the BCTs. Throughout the delivery period, the REPs and MHSWs will have four further half-day ‘supervision’ workshops to reflect on challenging and successful group discussions and to get expert and peer review of their intervention delivery.

#### Feasibility outcomes

The outcomes for this feasibility trial are:

- Referral rate recorded as the number of young people referred for screening via any route by the end of recruitment
- Recruitment rate recorded as the number of eligible participants who consent to participate in the study by the end of recruitment
- Attrition rate recorded as the number of participants who consent to participate that do not remain in the study until the end of follow-up at 26 weeks post randomisation
- Attendance rate at the intervention sessions as a proportion of the total number of sessions by 12 weeks
- Heart rate as measured using a heart rate monitor at each exercise session up to 12 weeks
- Physical activity measured using an accelerometer as proportion of time active at baseline, 14 weeks, and 26 weeks
- Adherence to the intervention protocol as captured by the intervention logs and rated against the adherence checklist by members of the study team at weekly intervention sessions and at 14 and 26 weeks
- Proportion of missing data will be reported as the percentage of recorded outcomes against those expected after account for withdrawal for each outcome separately at 26 weeks
- Adverse event rate recorded as the frequency, type (injury or clinical progression of depression), and severity of event by treatment arm at 26 weeks
- Estimate of resource use as measured through observation and study-specific questionnaire at 26 weeks
- Reach and representativeness measured by the proportions of young people who are screened for participation and are randomised in comparison to the characteristics of local populations by the end of recruitment

A traffic-light system, relating to recruitment, retention, adherence, and completion will inform whether we “stop”, “proceed”, or “proceed but with protocol changes” [64]. This system will be judged on criteria including young people’s attendance at sessions > 66% and questionnaire completion > 80% at 14 weeks. Additional process evaluation outcomes are the acceptability of the interventions and questionnaires, barriers and facilitators to engagement of young people, evaluating recruitment methods, and adherence to the intervention protocol by deliverers (fidelity).

#### Participant timeline

**Fig. 1:**
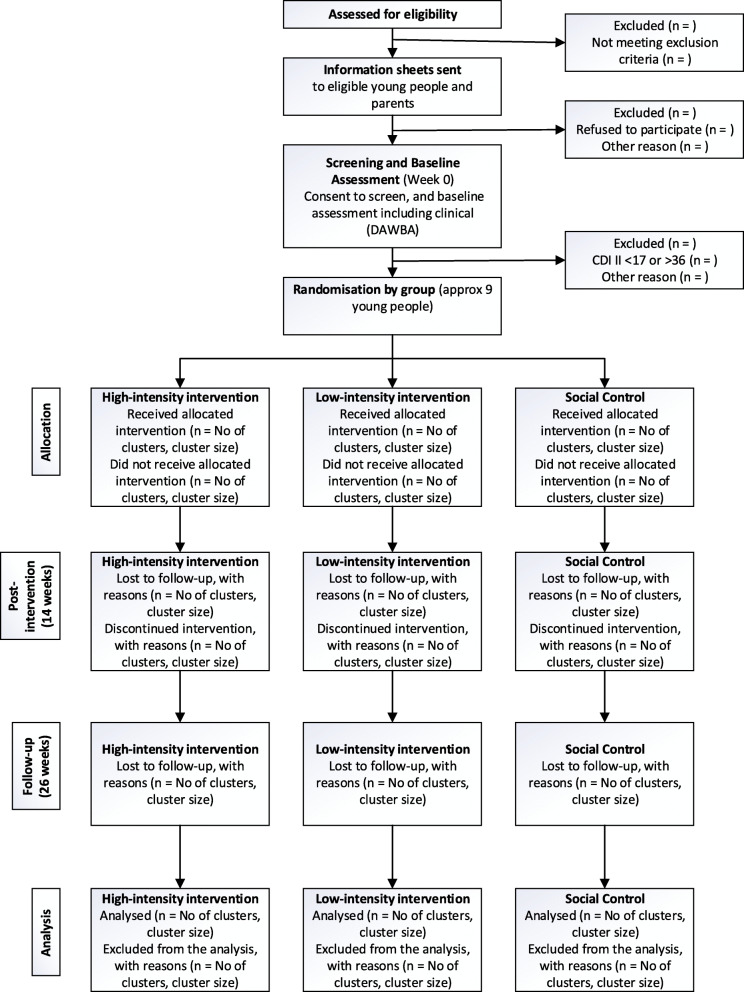
Study flowchart

Figure 1 shows the flowchart detailing study processes, highlighting the stages leading from participant eligibility assessment to final data analysis.

#### Sample size

Eighty-one eligible young people from three English counties (27/county; Hertfordshire, Bedford, and Norfolk) were selected. This sample size was selected to enable 20 participants per arm to attend the intervention sessions (assuming 33% dropout) and allows for each of the 3 interventions to be completed at each of the 3 study sites, giving 9 groups in total. Each group needs to have 9 participants to ensure that at any given session at least 6 participants are present (allowing for 33% no show/drop out). As this is a feasibility study it is not powered to detect a difference in clinical outcomes between the two intervention arms and the social control arm. Therefore, no power calculation has been performed. A sample size of 81 participants will allow us to gain experience in running each intervention at each site. It will also allow us to estimate the outcome completion rate to within ± 6% assuming a completion rate of 80% using an 80% confidence interval.

#### Recruitment

Young people will be recruited from Child and Adolescent Mental Health Services (CAMHS; Tiers 2 and 3), other Tier 2 services, and GP practices (Tier 1). Tier 2 services may be provided by NHS Trusts, but in some areas they are provided by the third sector (e.g. in Norfolk and Suffolk, Point 1 provide these services). The main organisation at each site is likely to be CAMHS (with responsibility to ensure clinical safety), with independent Tier 2 and GP practices acting as participant identification centres (PIC). The English counties involved are Hertfordshire (Hertfordshire Partnership University NHS Foundation Trust and Hertfordshire Community Trust), Norfolk (Norfolk & Suffolk NHS Foundation Trust), and Bedford (East London NHS Foundation Trust). These sites will be involved in recruitment to the study including screening and gaining consent from participants and collection of study outcome measures at baseline and follow-up. Effort was made to base the study across counties that would broaden the characteristics of the sample in terms of race/ethnicity and indices of deprivation.

### Assignment of interventions

Eligible participants who have consented to the study will be allocated to groups of approximately 9 participants, and each group will be randomised into one of the three trial arms (Table 3). This clustering is designed to limit waiting time for young people to start the intervention to minimise the dropout rate. Each group will either be a single-sex group or will include at least two girls and two boys. The former was deemed essential by patient and public involvement (PPI) representatives from diverse cultural heritage.

**Table 3:** Study schedule

	Study period						
	Screening	Randomisation	Post-randomisation				Post-randomisation follow-up
TIMEPOINT	Pre-T0	Pre-T0	T0	T0-T1	T1 +14 weeks	T2 +26 weeks	
**ENROLMENT:**							
**Eligibility screen**	X						
**Referral to research team**	X						
**Informed consent**	X						
**Randomisation**		X					
**INTERVENTIONS**				X			
**ASSESSMENTS:**							
***CDI-2***	X		X		X	X	
***Demographic information***	X						
***DAWBA (includes SDQ) CYP completed***	X						
***DAWBA (includes SDQ) Carer completed***	X						
***PAR-Q***	X						
***PANAS***			X		X	X	
***Self-efficacy scale***			X		X	X	
***Social Support Scale***			X		X	X	
***Caregiver burden***			X		X	X	
***Adherence***				X			
***Peak and average HR***				X			
***Ratings of Perceived Exertion***				X			
***Measured physical activity***			X		X	X	
***Y-PAQ***			X		X	X	
***COM-B measures***			X		X	X	
***EQ-5D-5L***			X		X	X	
***CSRI***			X		X	X	
***CHU-9D***			X		X	X	
***Focus groups***							X
**ADVERSE EVENT MONITORING**			X	X	X	X	X

Note: *FU* follow-up, *T0* baseline, *T1* first FU, *T2* second FU, *CDI-2* Clinical Depression Inventory – 2, *DAWBA* Development and Wellbeing Assessment, *SDQ* Strengths and Difficulties Questionnaire, *CYP* Child or Young Person, *PAR-Q* Physical Activity Readiness Questionnaire, *PANAS* Positive and Negative Affect Schedule, *Y-PAQ* Youth Physical Activity Questionnaire, *CSRI* Client Service Receipt Inventory, *CHU-9D* Child Health Utility 9D

#### Sequence generation

The allocated treatment for a group will be generated via computer written code using stratification with random permuted blocks. Stratification will be by study site.

#### Allocation concealment mechanism

The groups will be allocated to the intervention by a process embedded in the web-based data management system. When a group is randomised, an email will be sent to the principal investigator (PI), the Trial Manager, the MHSW, and the REP for them to set up the appropriate intervention.

#### Implementation

All participants who give consent for participation and who fulfil the inclusion criteria will be randomised. Randomisation will be performed centrally by the study manager using an online bespoke randomisation module provided and managed by Norwich Clinical Trials Unit (NCTU). The allocation sequence will be generated by the NCTU applying a permuted block design with random blocks stratified by study centre. The randomisation list remains with NCTU for the whole duration of the study. Thus, randomisation will be conducted without any influence of the principal investigators, MHSWs, or REPs.

#### Blinding (masking)

Due to the nature of the intervention, neither participants nor delivery staff can be blinded to allocation. However, it may be possible for the baseline and outcome assessments to be collected using a single blind (researcher) approach. Outcome assessment questionnaires will be completed online using mobile phones or tablets. However, it is anticipated that some young people may require assistance in completing the questionnaires, and therefore, we propose to evaluate the extent to which deliverers are needed to facilitate questionnaire completion at the 14- and 26-week follow-up sessions. Data will be analysed without access to information about allocation.

### Data collection, management, and analysis

#### Data collection methods

Screening data will be collected from patient notes and entered directly into the study database by members of the trial team working within each research site. Exceptionally, data may be entered onto paper case record forms prior to entry onto the database. Data collection, data entry, and queries raised by a member of the trial team will be managed in line with the NCTU and trial specific data management processes. Identification logs, screening logs, and enrolment logs will be kept at the trial site in a locked cabinet within a secured room. Patient reported screening data will be entered directly into the study database by the participant and their parent via an online questionnaire.

##### Baseline and follow-up

Young people and parents will be followed-up at 14 and 26 weeks. To maximise engagement with the young people in the study, baseline and follow-up data collection is planned as a group session (in trial groups), with the young people completing the outcome measures as online questionnaires either on their smartphones, or on tablets provided by the group facilitator. Individual follow-up via online questionnaire will be available where preferred by the young person. Caregiver burden data will be collected from parents via online survey, either at the group session or at home.

Attendance, adherence, and physical exertion data will be recorded on a paper form at each intervention session by the REP, and then entered into the study database immediately after the session has concluded. Heart rate data during the session will be recorded using chest band monitors and relayed to the REP’s tablet via Bluetooth. It will then be entered manually into the study database by the REP.

##### Outcome measures

*Psychological measures*

- Children’s Depression Inventory (CDI 2 [44];), which measures self-reported depressive symptoms in youth aged 7–17 years and takes 5–15 min to complete (primary outcome for the potential full trial)
- Positive and Negative Affect Schedule (PANAS [65];), with two 10-item sub-scales for positive and negative affect
- New General Self-Efficacy scale [66], an eight-item measure to assess how much people believe they can achieve their goals to engage in exercise
- Multidimensional scale of perceived Social Support (MSPSS [67];), a twelve-item measure designed to measure perceptions of support from family, friends, and a significant other
- A six-item COM-B measure asking participants how much they agree that they have the Capability (Physical and Psychological), Opportunity (Social and Physical), and Motivation (Reflective and Automatic) to be regularly active [68].

##### Carer measures

Burden Scale for Family Caregivers – short form (BSFC-s [69];) is a ten-item scale to measure perceived burden on families.

##### Physical activity

Peak and average heart rate (Polar, H10, Polar Electro, Kempele, Finland) and rating of perceived exertion during the intervention sessions [70]. Physical activity beyond the intervention sessions by accelerometer (GENEActiv Original, Activinsights, Cambridgeshire, UK) and the Youth Physical Activity Questionnaire (Y-PAQ [71];).

##### Trial intervention safety

For trial intervention safety, adverse events are recorded.

##### Economic evaluation

EQ-5D-5L, a measure of Health-Related Quality of Life (HRQoL [72];) uses both descriptive scales (five items) and a visual scale (EuroQol Visual Analogue System (EQ-VAS)); Child Health Utility 9D (CHU-9D [73];). A HRQoL questionnaire which will take approximately 5 min to complete. There are nine multiple-choice questions, each with a choice of five answers; modified client service receipt inventory (CSRI [74];) completed with the MHSW. If the young person is unable to complete this measure, then the parent/carer will be asked to provide missing information.

##### Process evaluation

A process evaluation will be conducted to identify and address key issues to inform and facilitate the main trial. It will examine intervention delivery and contamination between arms, barriers and facilitators to engagement of young people, and recruitment methods. The findings will be used to refine the intervention and study delivery for the main trial. Mixed methods used will include:

- Intervention logs completed by the REPs and MHSWs to examine intervention delivery and adherence, including numbers of young people attending, activities undertaken, and duration of each activity.
- Independent observations of 5–10% of the intervention sessions using an observation checklist to ascertain fidelity to the training.
- Focus groups with young people after the intervention sessions are completed (one focus group per site). Purposive sampling will be used to invite a diverse mix of young people to the focus groups. A semi-structured topic guide will be used to explore acceptability of the intervention and study methods, and barriers and facilitators to participation.
- Focus groups with the REPs and MHSWs involved in intervention delivery, in order to explore experiences of training and delivery (one focus group per site). A semi-structured topic guide will be used to explore experiences of intervention training and delivery.
- Case report forms will record young people’s reasons for declining to participate in order to inform recruitment strategies in the main trial. We will also record non-identifiable demographic information such as ethnicity and sex.

#### Data management

The database will assign participants a unique study participant identifier (PID) and all data will be entered against this number. The central database will be controlled and administered by NCTU Data Management. Features to help maintain data quality include the following: maintaining an audit trail; allowing users to raise data query requests; search facilities to identify validation failure/missing data.

#### Statistical methods

Data analysis for the feasibility study will be largely descriptive. The referral, recruitment (> 10% of potential young people recruited), and retention (> 66% attendance at intervention sessions) will be evaluated, reporting the proportions (and confidence intervals) of young people reaching each stage of the study, by referral source and study arm. Reach and representativeness will be described in relation to the proportions of young people who are screened for participation and are randomised, and in comparison, to the characteristics of local populations. Adherence will be assessed through the proportions of sessions attended and the average heart rate achieved compared to the target for the intervention. Behaviour change, in terms of increased physical activity beyond the intervention sessions, will be measured by accelerometer and self-report data at 14 and 26 weeks. Feasibility of collecting outcome and resource use data will be evaluated by estimating the proportions of missing data in each of the outcomes assessed. The outcome measures will be summarised, by study arm, using descriptive statistics based on the intention-to-treat principle. No formal comparison of the groups will be made. To monitor safety, the number of adverse events will be reported by study arm. 

### Process evaluation

Triangulation of the analysis of the intervention logs, focus groups, and case report forms will be used to describe and examine adherence and delivery of all three arms in order to refine the intervention for the full-scale trial. Intervention logs will be rated against the adherence checklist by members of the study team, to identify implementation fidelity and any potential contamination between arms. The focus groups will be transcribed verbatim and thematically analysed [75] using NVivo software. The findings from the focus groups with REPs and MHSWs will be synthesised with the intervention logs and independent observations of the sessions to explore adherence to training content and importantly to offer explanations for adherence and non-adherence. The findings from the focus groups with the young people will be considered along with the fidelity information from intervention logs and observations and quantitative adherence data (i.e. proportions of sessions attended and average heart rate). This will identify necessary modifications to the intervention for the main trial and to generate hypothetical propositions of the circumstances of successful delivery in the main trial.

We will examine reasons for declining to participate in the study and any demographic patterns amongst decliners to facilitate reach and representativeness of our recruitment strategy in the main trial. With reference to particular groups and local strategies outlined by Active Partnerships, recruitment strategies will be reviewed to aid our interpretation of recruitment figures for hard to reach groups of young people, which will then inform any recommended changes to recruitment strategy in the full-scale trial. This analysis will enable us to identify contextual factors that may affect adoption, delivery, and maintenance of the intervention.

### Economic evaluation

Resources required to provide interventions will be measured in all three groups. Resources measured will include the following: staff time; equipment and consumables; premises hire; and staff training. In addition to these resources, an effective intervention may affect the use of health and social care services, as well as costs borne by young people and families. These will be recorded by means of a modified CSRI. The young person will complete as much of this as they are able, in consultation with the MHSW. This would be supplemented with information from the parent/carer where the young person was unsure of any details. The time frame for the CSRI was the preceding 12 weeks. The design of this modified CSRI will be based on previous literature [73], and the intention is to make the questionnaire as simple as possible.

The main outcome of the economic evaluation will be quality-adjusted life years (QALYs). Two instruments capable of estimating QALYs will the used in the feasibility study; the EQ-5D-5L and CHU-9D, both collected at baseline, 14 weeks, and 26 weeks. It is intended to compare these two instruments. And secondly, by examining correlation with the CDI-2, this will inform a choice between the two in respect of the preferred choice for the full study. The analysis of health economics data will be largely descriptive. We will assess the completeness of these instruments and consider appropriate modifications for the main trial where indicated. Any resources identified by the CSRI will be costed using appropriate local and national cost data. This will enable the identification of key drivers of costs, and this will be used to inform the design of the CSRI in the main study.

### Monitoring

#### Data monitoring

The Data Monitoring and Ethics Committee (DMEC) will monitor the study data and make recommendations to the Trial Steering Committee (TSC) on whether there are any ethical or safety reasons why the trial should not continue. It will consider the need for any interim analysis and advise the TSC regarding the release of data and/or information. The TSC is comprised of a statistician, health economist, sports and exercise scientist, health improvement lead in public health, a GP, and specialists in behaviour change and PPI. The DMEC is comprised of a developmental psychopathologist, a statistician, and a psychiatrist.

#### Harms

When an adverse event (AE) occurs, the member of the study team who first becomes aware of the AE will assess whether the event is serious. If they are unsure of whether the event should be classified as serious, the team member will consult the local PI. All AEs assessed as non-serious, whether expected or not, will be recorded in the participant’s medical notes (if applicable) and recorded on the study database within 7 days. If it is apparent to any member of the study team that a number of AEs have been reported for one participant, they will refer this to the local PI who will review and escalate to the Trial Manager and Chief Investigator (CI), if necessary.

All serious adverse events (SAE) will be notified to the Trial Manager within one working day and an SAE form completed. This completed and signed SAE form should be emailed to the Trial Manager (or delegated person in the absence of the Trial Manager). The Trial Manager will review the SAE form and disseminate to the CI, PI, local R&D, the sponsor, and the appointed clinician who provides clinical oversight for the trial within 72 h of being informed. The DMEC and REC will be informed by the Trial Manager of SAEs periodically unless the CI or sponsor escalates the SAE or deems necessary. If an SAE is considered to be related to the trial intervention (based on a 5-point causality likelihood system: unrelated; unlikely to be related; possibly related; probably related; definitely related), and the intervention is discontinued or interrupted for that participant as a result, this will be recorded in the appropriate sections of the database. At the baseline visit, all participants, parents, and carers will be given information about accessing help locally (e.g. crisis team) should the mental health status of the young person deteriorate.

#### Auditing

Quality assurance activities will be conducted by the sponsor and NCTU. This will include an overarching review of key documents. Central and on-site monitoring will be conducted. Each site will be checked, with an on-site monitoring visit, at least once during the study. Central monitoring of the database will occur throughout the study.

### Patient and public involvement

Patient and public involvement (PPI) has been embedded within the study to ensure the relevance and appropriateness of study outputs for adolescents with depression and their families. We have involved young people, parents and carers, and members of the public in key discussions and decisions throughout the development of the study. This included consultation with a LGBTQ (lesbian, gay, bisexual, transgender, queer) group of young people (16–19 years old) and an ethnically mixed group of young women from Luton (aged 15–17 years) which helped us consider the complexity of gender in relation to exercise and depression, and to enhance inclusion and diversity in our PPI. Consultation with young people with depression and experience of CAMHS (aged 14–15 years) identified the importance of emotional support for young people participating in the study, leading to the addition of MHSWs to co-facilitate the activity groups, alongside the REPs. In addition, the Public Involvement in Research Group (PIRg) at the University of Hertfordshire provided advice to the team at different stages of study development and reviewed research ethics documents.

A key element of the embedded PPI in this trial is the establishment of a dedicated READY Young People’s Advisory Group (YPAG), consisting of 18 young people with lived experience of depression (personally or that of siblings/friends), whose views will be incorporated into the key decisions of the study. The YPAG will meet three times a year, be run in consultation with the young people themselves and aligned with examples of good practice of involving young people in research from NIHR INVOLVE [76].

We will recruit three parents or carers of young people with depression to the TSC from local parent/carer groups. PIRg members will also sit on the TSC and support the induction and training of the parents and carers and the two YPAG members, and to be PPI ‘mentors’. At different stages of the research, we will involve our PPI contributors in workshops for data analysis and interpretation, writing, and dissemination activities. We will record all PPI activity, meetings, and workshops so that we can report on the impact of the PPI in future reports and publications.

A stakeholder forum with representation from Mental Health Trusts, Active Partnerships (AP), Tier 2 Mental Health Services, Commissioners, General Practitioners, Public Health Teams, NHS England, NHS Improvement, and voluntary organisations will be held every 6 months. This will provide a way for the study team to communicate with the wider community, to follow policy development, to receive input into the design and delivery of the trials, and to support the dissemination programme. This forum will have input from PPI, including both the PIRg and YPAG. The study team, the TSC, and the DMEC will receive reports from the YPAG and from the Stakeholder forum.

### Ethics and dissemination

#### Protocol amendments

Although steps will be taken to avoid it, protocol deviations may happen subject to changes based on the evolution of the COVID-19 pandemic that was occurring during the time of writing. Protocol deviations that occur after the start of trial recruitment will be documented on a Protocol Deviation form and reported to the CI and Sponsor immediately. Deviations from the protocol which are found to frequently recur are not acceptable, will require immediate action, and could potentially be classified as a serious breach. Serious breaches to the principles of Good Clinical Practice and/or the protocol will be relayed to the sponsor immediately.

#### Consent or assent

Consent will be managed sensitively given the age of the young people. Participants will be provided sufficient time to read the information provided to them in the information sheet. They will be given the opportunity to ask questions they might have. If they are willing to continue, then consent/assent will be given. The parents or carers will be consented to provide study assessment about the young person in their care (this is an inclusion criteria). Young people aged 16 or older will provide written consent, and those under 16 will be asked to provide written assent to participate but will also require consent to be provided by their parent or carer. During this feasibility phase, the numbers of young people unable to join the study for this reason will be recorded. In some cases, the role of the parent may be taken by a legal carer (foster carer, or Social Services), and in these cases, relevant consent and engagement by carers (sometimes not the legal guardian) will be facilitated.

#### Confidentiality

All study-related information will be stored securely at each study site. All participant information will be stored in locked file cabinets. All reports, data collection, process, and administrative forms will be identified by a coded ID number only to maintain participant confidentiality. All records that contain names or other personal identifiers will be stored separately from study records identified by a code number. All local databases will be secured with password-protected access systems. Any documents that link participant ID numbers to other identifying information will be stored in a separate, locked file.

### Access to data

Access to the database will be managed by NCTU and will be restricted to authorised personnel and password protected. The audit trail will be monitored regularly for any unauthorised access. It is the responsibility of the CI/PI to ensure that relevant personnel are delegated to carry out data collection and data entry. The delegation log will identify all personnel with responsibilities for data collection and handling. After completion of the study, the database will be retained on the servers of NCTU for ongoing analysis of secondary outcomes, and the study database and associated design documentation will be routinely archived for a period of 10 years.

### Ancillary and post-trial care

Participants will be able to access NHS services as necessary during the study without restriction. There is no provision for the intervention to be available post-trial. An option post-trial is for all participants irrespective of study allocation to be offered signposting to local exercise groups and facilities.

### Dissemination policy

The primary output will be the report at the end of this feasibility phase which will recommend whether the main study can proceed and inform the design of that study. The results will also be disseminated by the NIHR and through high-impact international peer-reviewed journals, the HTA/NIHR, and at conferences for clinicians, commissioners, and researchers working in CAMHS, primary care, public health, Local Authorities, Active Partnerships and Sports/Football community trusts, and voluntary organisations. Patient and public involvement will be embedded in the study, and with input, we will develop a project website and social media presence to inform and engage young people and their families, clinicians, NHS providers and commissioners, and the wider population about the progress, findings and impact of our research. Our YPAG will help develop meaningful and appropriate ways of telling young people about our research, based on previous research conducted with young people with depression [77].

### Trial status

Screening and recruitment was provisionally due to run from March 2020 to August 2020. Due to the COVID-related delays, the approved revised plan is for recruitment to run from September to December 2020, the interventions sessions to run from October 2020 to April 2021, follow-up outcomes to be captured between January and July 2021, data to be analysed from June to November 2021, and write-up to span October to November 2021. The amended plan will depend on ongoing developments and government advice related to COVID-19, but the trial team have developed an online delivery alternative that will mean the intervention can continue should restrictions be heightened again. Version number 2.0, 30th July 2020.

## Data Availability

Not applicable, no datasets are included in this study protocol.

## Abbreviations

AE: Adverse event
BAME: Black Asian and minority ethnicity
BCT: Behaviour change techniques
BSFC-s: Burden Scale for Family Caregivers – short form
CAMHS: Child and Adolescent Mental Health Services
CDI: Clinical Depression Inventory
CHU-9D: Child Health Utility 9D
CONSORT: Consolidated Standards of Reporting Trials
CTU: Clinical trials unit
CSRI: Client Service Receipt Inventory
DMEC: Data Monitoring and Ethics Committee
DAWBA: Development and Well-Being Assessment
EQ-VAS: EuroQol Visual Analogue Scale
HIIT: High-intensity interval training
HRQoL: Health-related quality of life
HTA: Health technology assessment
LGBTQ: Lesbian, gay, bisexual, transsexual, queer
MHSW: Mental health support worker
MSPSS: Multidimensional Scale of Perceived Social Support
NICE: National Institute for Health and Care Excellence
NIHR: National Institute for Health Research
NETSCC: NIHR Evaluation Trials and Studies Co-ordinating Centre
OARS: Open-ended questions, Affirmations, Reflective listening, Summaries
PANAS: Positive and Negative Affect Schedule
PAR-Q: Physical Activity Readiness Questionnaire
PI: Principal investigator
PIC: Participant identification centre
PID: Participant identifier
PIRg: Public Involvement in Research Group
PPI: Patient and public involvement
RCT: Randomised controlled trial
REC: Research Ethics Committee
REP: Registered exercise professionals
R&D: Research & Development Department
RULE: Resist the righting reflex; Understand your client’s motivation; Listen to your client; Empower your client)
SAE: Serious adverse event
TDF: Theoretical Domains Framework
TMG: Trial Management Group
TSC: Trial Steering Group
UK: United Kingdom
